# Impact of α-Linolenic Acid, the Vegetable ω-3 Fatty Acid, on Cardiovascular Disease and Cognition

**DOI:** 10.1093/advances/nmac016

**Published:** 2022-02-16

**Authors:** Aleix Sala-Vila, Jennifer Fleming, Penny Kris-Etherton, Emilio Ros

**Affiliations:** Fatty Acid Research Institute, Sioux Falls, SD, USA; Cardiovascular Risk and Nutrition, Hospital del Mar Medical Research Institute (IMIM), Barcelona, Spain; Department of Nutritional Sciences, College of Health and Human Development, Pennsylvania State University, University Park, PA, USA; Department of Nutritional Sciences, College of Health and Human Development, Pennsylvania State University, University Park, PA, USA; Lipid Clinic, Endocrinology and Nutrition Service, Hospital Clínic, August Pi i Sunyer Biomedical Research Institute (IDIBAPS), Barcelona, Spain; CIBER Physiopathology of Obesity and Nutrition (CIBEROBN), Instituto de Salud Carlos III (ISCIII), Madrid, Spain

**Keywords:** alpha-linolenic acid, long-chain n–3 fatty acids, cardiovascular disease risk, lipids, blood pressure, flaxseed, walnuts

## Abstract

Given the evidence of the health benefits of plant-based diets and long-chain n–3 (ω-3) fatty acids, there is keen interest in better understanding the role of α-linolenic acid (ALA), a plant-derived n–3 fatty acid, on cardiometabolic diseases and cognition. There is increasing evidence for ALA largely based on its major food sources (i.e., walnuts and flaxseed); however, this lags behind our understanding of long-chain n–3 fatty acids. Meta-analyses of observational studies have shown that increasing dietary ALA is associated with a 10% lower risk of total cardiovascular disease and a 20% reduced risk of fatal coronary heart disease. Three randomized controlled trials (RCTs) [AlphaOmega trial, Prevención con Dieta Mediterránea (PREDIMED) trial, and Lyon Diet Heart Study] all showed benefits of diets high in ALA on cardiovascular-related outcomes, but the AlphaOmega trial, designed to specifically evaluate ALA effects, only showed a trend for benefit. RCTs have shown that dietary ALA reduced total cholesterol, LDL cholesterol, triglycerides, and blood pressure, and epidemiologic studies and some trials also have shown an anti-inflammatory effect of ALA, which collectively account for, in part, the cardiovascular benefits of ALA. A meta-analysis reported a trend toward diabetes risk reduction with both dietary and biomarker ALA. For metabolic syndrome and obesity, the evidence for ALA benefits is inconclusive. The role of ALA in cognition is in the early stages but shows promising evidence of counteracting cognitive impairment. Much has been learned about the health benefits of ALA and with additional research we will be better positioned to make strong evidence-based dietary recommendations for the reduction of many chronic diseases.

## Teaser

**Statement of Significance**: This narrative review updates the evidence from epidemiologic studies, randomized controlled trials, and meta-analyses about dietary α-linolenic acid and cardiovascular disease and its intermediate risk factors, as well as metabolic syndrome, type 2 diabetes, and cognition.

## Introduction

α-Linolenic acid (ALA; 18:3n–3) is an essential fatty acid (1). ALA is a component of oleaginous seeds and oils, as well as nuts (particularly walnuts), and is the only source of omega-3 (n–3) PUFAs in the diet of vegans and one of the only dietary sources for vegetarians [eggs contain small quantities of DHA (22:6n–3)] (2). The conversion of ALA to EPA (20:5n–3) is limited (particularly in men) and further conversion to DHA is marginal (3). Previous research on the health benefits of dietary EPA and DHA has obscured the role of ALA. However, ALA has recently been the focus of much scientific interest. Because many individuals avoid fish for different reasons (taste preferences, vegetarianism/veganism, sustainability sensitivities, and concern about mercury and other contaminants) and there is growing pressure on fisheries to meet consumption demands, there has been a concerted effort to better understand the beneficial properties of dietary ALA independent of marine-derived n–3 PUFAs. In this narrative review, we will update the evidence from epidemiologic studies, randomized controlled trials (RCTs), and meta-analyses concerning exposure to ALA and cardiovascular disease (CVD) [reviewed in 2014; (4)]; we also will consider evidence on intermediate risk factors, as well as cardiometabolic risk markers, including type 2 diabetes (T2D), metabolic syndrome (MetS), and obesity. The final section addresses cognition.

We will review the epidemiologic evidence based on self-reported dietary ALA (mostly from FFQs) and chemically determined ALA in adipose tissue or blood (as biomarker of ALA intake). Because ALA is an essential fatty acid, dietary ALA intake has been shown to correlate well with the ALA content in adipose tissue and all blood lipid fractions in both observational studies and RCTs (5). For RCTs, we review dietary supplementation with refined ALA or foods rich in ALA, i.e., flaxseed oil, canola (rapeseed) oil, or walnuts as a food, oil, or spread. For both epidemiologic studies and RCTs, data from meta-analyses are summarized in tabular form, whereas relevant individual studies not included in meta-analyses are discussed separately. Finally, we propose recommended intakes for ALA and discuss research needed to make evidence-based recommendations for ALA for cardiovascular and brain health.

## Current Status of Knowledge

### ALA and CVD

The 2020–2025 Dietary Guidelines for Americans (6) concluded there is strong evidence to demonstrate that replacing SFAs with PUFAs reduces the risk of ischemic heart disease (IHD) events and CVD mortality. Moreover, moderate evidence indicates that intake of n–3 PUFAs, particularly the marine-derived long-chain n–3 PUFAs (LCn–3PUFAs) EPA and DHA, is associated with a lower risk of CVD (7). However, there remains a gap in the evidence about the benefits of ALA apart from LCn–3PUFAs on CVD outcomes. Current recommendations for ALA are based on an Adequate Intake and have remained at 1.1–1.6 g/d (6) despite evidence suggesting cardiovascular benefits at amounts >2 g/d (0.6%–1% total energy). Several recent publications and previous landmark trials suggest that increasing dietary ALA may confer additional benefits (7). Importantly, however, there are many inconsistencies in the literature, attributable to factors such as duration of the study or supplementation period, dose, gender, ethnicity, and habitual diet. Additional limitations of these findings are the variability among studies in the source of ALA provided, e.g., capsules containing oil, seed oils, or whole foods, all of which influence the availability of ALA.

#### Total CVD

##### Epidemiologic evidence—self-reported ALA intake

In a 2012 meta-analysis of observational studies (8), increasing dietary ALA was associated with a moderately lower risk of total CVD (RR: 0.90; 95% CI: 0.81, 0.99) (**Table 1**). To better understand the role of ALA independent of EPA and DHA, Sala-Vila et al. (9) prospectively evaluated the association between dietary ALA and fatal CVD in participants of the PREvención con DIeta MEDiterránea (PREDIMED) study (*n* = 7202). No association was observed for cardiovascular mortality, but dietary ALA at ≥0.7% of daily energy intake was associated with a 28% reduced risk of all‐cause mortality, with a further reduction in participants reporting an intake of EPA + DHA ≥500 mg/d. This finding suggests that intake of both marine and plant-derived n–3 PUFAs may act synergistically and not competitively, at least concerning all-cause mortality (9). In the Singapore Chinese Health Study (890,473 person-years of follow-up, 4780 deaths), decreased odds of cardiovascular mortality were observed for both intake of LCn–3PUFAs (top quartile HR: 0.86; 95% CI: 0.77, 0.96) and ALA (HR: 0.81; 95% CI: 0.73, 0.90). After stratification for type of event, results were maintained for fatal IHD but not for fatal stroke (10). However, in a large Danish cohort (*n* = 53,909), a significant inverse association between ALA intake and the rate of atherosclerotic CVD was reported in individuals with intakes of LCn–3PUFAs <10th percentile (<0.252 g/d); no association was observed among participants with intakes >10th percentile (11).

**TABLE 1:** Meta-analyses of epidemiologic studies reporting associations between dietary intake or biomarker ALA and health outcomes^1^

Authors (ref.)	Outcomes assessed	Numbers in meta-analysis	Follow-up of cohort studies, y	Source of ALA, *n* studies	HRs/ORs/RRs (95% CIs) for comparisons of extreme quantiles or linear increases of ALA
Pan et al. (8)	Total CVD	27 prospective and case-control studies; *n* = 251,049; 15,327 events	5–31	Dietary intake (*n* = 10)Biomarker (*n* = 14)Both (*n* = 3)	Dietary studies: RR: 0.90 (0.81, 0.99)Biomarker studies: RR: 0.80 (0.63, 1.03)Overall: RR: 0.86 (0.77, 0.97)
	Fatal IHD	9 studies		Dietary intake (*n* = 6)Dose-response (*n* = 5)Biomarker (*n* = 3)	Dietary studies: RR: 0.80 (0.65, 0.98)Per 1-g/d increment: RR: 0.90 (0.83, 0.99)Biomarker studies: RR: 1.07 (0.66, 1.75)
	Nonfatal IHD	10 studies		Dietary intake (*n* = 3)Biomarker (*n* = 7)	Dietary studies: RR: 0.84 (0.61, 1.15)Biomarker studies: RR: 0.81 (0.60, 1.09)
	Stroke	5 studies		Dietary intake (*n* = 3)Biomarker (*n* = 2)	Dietary studies: RR: 0.96 (0.78, 1.17)Biomarker studies: RR: 0.77 (0.37, 1.60)
Harris et al. (17)	CVD mortalityAll-cause mortality	17 prospective studies; *n* = 42,466; 15,720 total deaths; 4571 CVD deaths	5–32	Biomarker (*n* = 17)	All-cause mortality: HR: 0.99 (0.96, 1.02)CVD mortality: HR: 1.01 (0.95, 1.07)
Wei et al. (22)	Total IHDIHD mortality	14 prospective studies;*n* = 345,202	4–22	Dietary intake (*n* = 14)	Total IHD: RR: 0.91 (0.85, 0.97)Fatal IHD: RR: 0.85 (0.75, 0.96)
Del Gobbo et al. (33)	Total IHDIHD mortality	19 prospective studies; *n* = 45,637;7973 total IHD cases; 2781 fatal IHD cases	18–97	Biomarker (*n* = 19)	Total IHD (per 1-SD/d increase): RR: 1.00 (0.95, 1.05)Nonfatal IHD (per 1-SD/d increase): RR: 0.95 (0.87, 1.05)Fatal IHD (per 1-SD/d increase): RR: 0.91 (0.84, 0.98)
Wu et al. (101)	T2D	16 prospective studies; *n* = 540,184; 25,670 incident cases	4–17	Dietary intake (*n* = 7)Biomarker (*n* = 6)	Dietary studies: RR: 0.93 (0.83, 1.04)Biomarker studies: RR: 0.90 (0.80, 1.00)
Neuenschwander et al. (102)	T2D	23 prospective studies;*n* = 450,568 for LCn–3FAs (24,396 incident cases) and *n* = 242,676 for ALA (13,499 incident cases)	4–32	Dietary intake (*n* = 11)	Per 250-mg/d increase: RR: 1.01 (0.98, 1.05)
Qian et al. (103)	T2D	20 prospective studies; *n* = 65,147;16,693 incident cases	3–21	Biomarker (*n* = 20)	HR: 0.97 (0.92, 1.02)
Guo et al. (106)	MetS	15 cross-sectional and case-control studies	Not applicable	Biomarker (*n* = 5)	OR: 1.58 (0.89, 2.82)
Jang and Park (107)	MetS	13 studies, mostly cross-sectional;*n* = 36,542	Not applicable	Biomarker (*n* = 6)Dietary intake (*n* = 1)	OR: 1.22 (0.84, 1.76)

1 ALA, α-linolenic acid; CVD, cardiovascular disease; HR, hazard ratio; IHD, ischemic heart disease; LCn–3FA, long-chain n–3 fatty acid; MetS, metabolic syndrome; OR, odds ratio; RR, risk ratio; T2D, type 2 diabetes.

##### Epidemiologic evidence—biomarker

The 2012 meta-analysis by Pan et al. (8) included cohorts that used an ALA biomarker as the exposure and reported a nonsignificant trend toward a lower risk of incident CVD (Table 1). Several longitudinal studies on the topic have appeared since then (12–16) and were included in a recent comprehensive meta-analysis utilizing a harmonized analytical strategy with individual-level data from 17 cohorts, which reported that LCn–3PUFA species, but not ALA, were associated with moderately reduced total and CVD mortality (Table 1) (17). Finally, each 1-SD increment in serum phosphatidylcholine EPA at hospital admission for ST-segment-elevation myocardial infarction (MI) (*n* = 944) was associated with a ∼25% lower rate of both incident major cardiovascular events and readmission for cardiovascular causes after 3 y of follow-up. ALA in phosphatidylcholine showed a trend toward lower incident cardiovascular events but was inversely related to all-cause mortality (HR: 0.65; 95% CI: 0.44, 0.96) (18). In another study in non-ST-segment-elevation acute coronary syndrome patients, increasing proportions of plasma LCn–3PUFAs were associated with a lower risk of cardiovascular death (OR: 0.82; 95% CI: 0.68, 0.98 per 1-SD increment), whereas an attenuated relation was observed for ALA (OR: 0.92; 95% CI: 0.74, 1.14) (19).

##### RCTs

Few RCTs have specifically evaluated dietary sources of ALA and CVD-related outcomes. In the 3.3-y AlphaOmega trial, examining 4 interventions (1.9 g/d of ALA, 400 mg/d of EPA + DHA, both, or placebo) in 4837 MI survivors, the primary endpoint was the rate of major cardiovascular events, which comprised fatal and nonfatal CVD and cardiac interventions. When combining the 2 groups that received margarine containing ALA (alone or with EPA + DHA), there was a nonsignificant reduction in CVD incidence (HR: 0.91; 95% CI: 0.78, 1.05) (20).

In the PREDIMED RCT of a Mediterranean diet intervention for the primary prevention of CVD, participants (*n* = 7447) at high CVD risk in 1 treatment arm who received 30 g/d of mixed nuts (15 g walnuts, 7.5 g hazelnuts, and 7.5 g almonds) had a reduced incidence of cardiovascular events (HR: 0.72; 95% CI: 0.54, 0.95) after 4.8 y compared with the control group (advice to follow a low-fat diet). In the nuts arm, dietary ALA increased by 0.43 g/d and plasma ALA increased by 0.30%–0.44% in a random subsample. The benefit could not be ascribed only to ALA, because nuts contain other bioactives and participants were advised to increase adherence to the Mediterranean diet (21).

#### IHD

##### Epidemiologic evidence—self-reported ALA intake

Numerous studies have examined associations between dietary ALA and IHD risk. The meta-analysis by Pan et al. (8) found that higher compared with lower ALA intake was associated with reduced risk of fatal IHD (RR: 0.80; 95% CI: 0.65, 0.98), but not of nonfatal IHD or total IHD (Table 1). Also, in dose-response analyses, each additional 1 g/d of ALA intake was associated with a 10% reduction in risk of fatal IHD (RR: 0.90; 95% CI: 0.83, 0.99). A 2018 updated meta-analysis of 14 cohort studies (22) reported modest associations between higher ALA intake and lower risk of total IHD and fatal IHD (Table 1).

An investigation of 8 prospective cohort studies by Vedtofte et al. (23) reported a tendency toward effect modification by gender of the association of ALA with IHD death (*P*-interaction = 0.07). In men (*n* = 80,368) a nonsignificant inverse association was observed between the intake of ALA and the risk of IHD events and deaths. For each additional gram of ALA consumed, a 15% lower risk of IHD events (HR: 0.85; 95% CI: 0.72, 1.01) and a 23% lower risk of IHD death (HR: 0.77; 95% CI: 0.58, 1.01) were observed. No consistent associations were observed among women (*n* = 148,675). However, no between-gender differences were observed for incident MI in a Danish cohort study (11). Why the ALA results may differ between women and men remains unclear. There is increasing evidence of a sexual dimorphism on risk factor responses to dietary interventions in favor of men (24–31). In addition to gender, it has been postulated that EPA influences ALA metabolism via feedback inhibition of the activity of δ-6-desaturase, the key limiting enzyme regulating the conversion of ALA to LCn–3PUFAs. Therefore, associations between dietary ALA and IHD would be stronger in the presence of low marine n–3 PUFA intakes. This was suggested by the Health Professionals Follow-Up Study, in which an inverse association between intake of ALA and the risk of IHD among US men with low LCn–3PUFA intake (<100 mg/d) was reported. An increase of 1 g/d of ALA was associated with a 58% lower nonfatal MI risk (HR: 0.42; 95% CI: 0.23, 0.75) and 47% lower total IHD risk (HR: 0.53; 95% CI: 0.34, 0.83) among men with low EPA + DHA intake, whereas no association was reported among those with higher LCn–3PUFA intake (32). In the analysis of 8 prospective cohort studies by Vedtofte et al. (23), among men with LCn–3PUFA intakes below the median (<0.26 g/d), each 1 g/d of ALA intake was associated with a 27% lower risk of IHD (HR: 0.73; 95% CI: 0.51, 1.04), whereas no significant association was observed in those above the median (HR: 0.90; 95% CI: 0.72, 1.12); no effect modification by LCn–3PUFA intake was observed among women.

##### Epidemiologic evidence—biomarker

The meta-analysis by Pan et al. (8) reported no associations between ALA biomarker and fatal or nonfatal IHD (Table 1). In the Cardiovascular Health Study there was no evidence that a higher proportion of ALA in plasma phospholipids at baseline was associated with a change in IHD mortality (13). A pooling project of 19 cohort studies from 16 countries and measures of circulating or tissue biomarkers of n–3 PUFAs allowed for the separate investigation of each n–3 PUFA (33). In continuous analyses, ALA was associated with a 9% lower risk of fatal IHD, but was unrelated to total or nonfatal IHD (Table 1). In a case-control substudy of the Singapore Chinese Health Study, plasma ALA (highest compared with lowest quintile) was marginally associated with a lower risk of acute MI (OR: 0.73; 95% CI: 0.51, 1.05), whereas the association for LCn–3PUFAs was stronger (OR: 0.62; 95% CI: 0.41, 0.94) (34). Finally, a recent case-cohort study in patients with acute coronary syndrome found no significant associations of plasma ALA with subsequent MI or arrhythmic events (18).

##### RCTs

The Lyon Diet Heart Study was a landmark RCT that demonstrated the effectiveness of a Mediterranean-type diet supplemented with ALA on composite measures of IHD recurrence after a first MI (35). Participants in the experimental group received instructions to follow a Mediterranean-style diet and were given a canola oil–based margarine containing 4.8% ALA, which provided ∼0.5 g/d. After 46 mo, the experimental group had a 50%–70% lower risk of recurrent IHD (36). When analyzing plasma fatty acids as crude estimates of dietary data, only ALA was significantly associated with an improved prognosis (RR for the composite of cardiac death and nonfatal acute MI: 0.20; 95% CI: 0.05, 0.84). However, as in the PREDIMED trial, the benefit could not be ascribed entirely to ALA because other dietary changes across groups occurred. In the large AlphaOmega trial, after 40 mo neither ALA nor EPA + DHA reduced the prespecified endpoint of fatal IHD (20).

#### Stroke

##### Epidemiologic evidence—self-reported ALA intake and biomarker

The meta-analysis by Pan et al. (8) reported no significant associations between ALA assessed by FFQ or as a biomarker and stroke (Table 1). Since then, few studies have investigated the association between ALA intake and stroke, with inconclusive results. No association of ischemic stroke with serum n–3 PUFAs, either ALA or LCn–3PUFAs, was observed in 3870 men from the Atherosclerosis Risk in Communities cohort with a median follow-up of 19.9 y (37). In a case-control study nested in the Women's Health Initiative observational cohort of postmenopausal US women, both serum docosapentaenoic acid (DPA; 22:5n–3) and DHA (but not ALA) were inversely associated with incident stroke (38). In the Cardiovascular Health Study, no significant associations of plasma phospholipid ALA (*n* = 2709 participants) or self-reported dietary ALA (*n* = 2583 participants) were observed with the risk of stroke (13). In a substudy conducted in 1828 men from the prospective, population-based Kuopio Ischaemic Heart Disease Risk Factor Study, no significant associations were observed for any n–3 PUFA in serum and total, ischemic, or hemorrhagic stroke (39). In the Women's Health Study (*n* = 39,876; age ≥ 45 y), dietary ALA intake at baseline was not associated with incident total or ischemic stroke (40). Finally, 2 complementary studies reported findings from the Danish case-cohort study. Dietary ALA (*n* = 57,053; follow-up: 13.5 y) was unrelated to the risk of ischemic stroke or stroke subtypes (41). When considering ALA in adipose tissue (*n* = 3500; follow-up: 13.4 y), there was a statistically significant U-shaped association between ALA and the rate of ischemic stroke due to large artery atherosclerosis, with the lowest rate observed around the median content of adipose tissue ALA (42).

Three studies focused on structural brain alterations intimately related to stroke risk. In 1 study, DHA in plasma phospholipids, but not ALA, was found to be associated with lower risk of brain MRI-assessed prevalent subclinical lacunar infarcts, better white matter grade, and lower risk of worsening white matter. ALA was directly associated with better sulcal grade and ventricular grade. Results were unaffected when replacing ALA in phospholipids with self-reported dietary ALA (43). In an aging project involving 282 brain donors, the authors found decreased odds of cerebral macroinfarcts and microinfarcts in those who self-reported higher dietary ALA intake first measured by an FFQ at a mean of 4.5 y before death (44). Finally, in a recent cross-sectional study of 1657 patients with atrial fibrillation (AF) who underwent brain MRI, circulating EPA (but not DHA or ALA) was associated with a lower prevalence of ischemic brain infarcts (45).

##### RCTs

No RCTs of n–3 PUFAs specifically for stroke have been conducted. The PREDIMED trial showed that the Mediterranean diet supplemented with mixed nuts (containing 15 g/d of walnuts) resulted in a significant 45% reduction of incident stroke as an isolated endpoint, whereas there were nonsignificant trends for lower MI and CVD mortality (21).

#### Heart failure

##### Epidemiologic evidence—self-reported ALA intake and biomarker

Several prospective studies have assessed ALA and incident heart failure (HF). Two studies reported dietary data only. The first was conducted in 84,493 participants from the Women's Health Initiative observational cohort. Baseline intake of neither ALA nor EPA + DHA was related to the risk of incident HF during 10 y of follow-up (46). Similarly, no significant associations with HF were observed for baseline intake of ALA in 36,234 Swedish Mammography Cohort participants followed for >9 y (47).

One of the first studies on biomarker ALA and incident HF was conducted in the Atherosclerosis Risk in Communities cohort (*n* = 3592; age: 45–64 y; follow-up: 14.3 y). Whereas circulating DHA was found to be inversely associated with the risk of incident HF in women, no significant results were observed for ALA (48). Similarly, a substudy involving nearly 3000 participants from the Cardiovascular Health Study found that increasing plasma phospholipid EPA, DPA, and DHA were related to a significantly decreased risk of incident HF (49), a finding not observed for ALA (50). Results remained unchanged when considering dietary ALA as the exposure (50). In contrast to previous findings, in the Physicians’ Health Study, the authors reported an inverse and nonlinear relation of plasma phospholipid ALA (but not EPA or DHA) with HF risk, a result that was not observed for dietary ALA (51).

##### RCTs

No RCTs evaluating the effects of ALA on HF are available. The only RCT evidence of n–3 PUFAs is on N-terminal-pro brain natriuretic peptide, a biomarker of HF, in a randomly selected subsample of the AlphaOmega trial in patients with prior MI. Neither ALA nor EPA + DHA had any significant effect on this parameter (52).

#### Arrhythmia

##### Epidemiologic evidence—self-reported ALA intake and biomarker

Two prospective studies assessing ALA intake using dietary records or plasma biomarkers for the outcome AF reported no association (53, 54). Similarly, a recent case-cohort study in patients with acute coronary syndrome found no significant associations of plasma biomarkers of ALA or LCn–3PUFAs with AF (19). Likewise, findings from 3 large Scandinavian prospective cohorts do not support an association between LCn–3PUFA intake and incident AF (55, 56). Whereas ALA does not appear to affect atrial arrhythmia, there is evidence from a single cross-sectional study in 260 patients with MI that greater intake of n–3 PUFAs, both marine and ALA, is associated with lower ventricular premature beats, suggesting a reduced risk of ventricular arrhythmias (57).

##### RCTs

Only 2 large trials have evaluated ALA supplementation for cardiovascular outcomes, including arrhythmias. In the AlphaOmega trial, in post hoc analyses in the subgroup of patients with diabetes, who are particularly prone to ventricular arrhythmias and sudden death after MI, ALA compared with placebo or EPA + DHA led to a significant reduction of arrhythmia-related events (HR: 0.39; 95% CI: 0.17, 0.88). However, the rates of both fatal IHD and arrhythmia-related events were lower in the EPA + DHA groups (than in those receiving no EPA + DHA) (20). Another report from the AlphaOmega trial with 1014 diabetic participants showed a significant reduction of ventricular arrhythmia–related events for the EPA + DHA plus ALA group compared with placebo (HR: 0.16; 95% CI: 0.04, 0.69), pointing to an additive antiarrhythmic effect of plant-derived and marine n–3 PUFAs (58). The results of the AlphaOmega trial suggest that ALA intake has an antiarrhythmic effect, but more clinical trials are needed to confirm this. The Lyon Diet Heart study, in which an ALA-enriched margarine was given to the intervention group, had too few arrhythmic events to obtain meaningful information (36). Finally, FLAX-PAD, a small RCT (*n* = 110) testing whether daily consumption of 30 g milled flaxseed (or placebo) over 1 y modified the prevalence of cardiac arrhythmias and exercise capacity in patients with peripheral artery disease, did not detect significant between-group changes (59).

With regard to RCTs of LCn–3PUFA supplementation, a recent comprehensive meta-analysis concluded with low-certainty evidence that LCn–3PUFA intake may slightly increase the risk of arrhythmia in general (60). Of greater concern, the results of a recent meta-analysis of large trials using marine n–3PUFAs in patients with elevated plasma triglycerides (TGs) and at high CVD risk suggest that this supplementation is associated with an increased risk of AF, with a 5-trial summary HR of 1.37 (95% CI: 1.22, 1.57) compared with placebo (61). Although this adverse effect of LCn–3PUFAs is counterintuitive and lacks a reasonable explanation, this is in contrast to the null effect of ALA on AF and its suggested beneficial effect on ventricular arrhythmia.

### ALA and CVD risk markers

There is abundant evidence on the effects of ALA on different CVD risk factors. Most studies, mainly RCTs, have focused on lipids/lipoproteins along with effects on blood pressure (BP), inflammatory markers, and other cardiometabolic disease indexes. The source of ALA has varied, ranging from supplements to different food sources. In some of these studies, comparisons were made between marine-derived and plant-based n–3 PUFAs. The evidence indicates that ALA has beneficial effects on CVD risk markers. The research to date is increasing our understanding of the potential mechanisms by which ALA benefits CVD risk.

#### Dyslipidemia

A recent cross-sectional study examined associations between PUFA intake and the risk of dyslipidemia in a representative sample of US adults from NHANES 2009–2016 (62). The daily intakes of DHA, EPA, DPA, and ALA (and also stearidonic acid, another plant-derived n–3 PUFA) were based on the average of two 24-h dietary recalls. When grouped into tertiles, PUFA intake > 19.5 g/d was associated with an independent 19% decrease in dyslipidemia risk (OR: 0.81; 95% CI: 0.71, 0.94) compared with the lowest tertile (PUFA intake ≤ 12.3 g/d). A weaker association was observed for ALA + stearidonic acid (OR: 0.87; 95% CI: 0.76, 0.99). The protective association between total PUFAs and dyslipidemia appeared to reach a threshold at ∼19 g/d, but no threshold was observed for ALA + stearidonic acid. The authors postulate the difference could be related to pollutants in fish, the main source of LCn–3PUFAs in the US diet, which is of lesser concern for food sources of ALA (62).

The effects of marine n–3 PUFAs on plasma lipids have been studied extensively. For hypertriglyceridemia, 4 g/d of EPA + DHA decreases TGs by ≥30% with a concurrent increase in LDL cholesterol; isolated EPA, however, does not raise LDL cholesterol (63). With regard to ALA, a 2009 meta-analysis of 28 RCTs of flaxseed interventions for lipid outcomes showed modest reductions of total cholesterol and LDL cholesterol (**Table 2**), which were more pronounced in women and in individuals with high initial cholesterol concentrations (64). No effect was demonstrated for TGs and HDL cholesterol. Several previous RCTs have compared EPA/DHA and ALA on lipid/lipoprotein concentrations and reported inconsistent findings. A recent meta-analysis of RCTs compared the effects of ALA and EPA/DHA supplementation with placebo on cardiometabolic risk factors (65). Participants in the ALA arms had a modest LDL cholesterol reduction compared with placebo, whereas those in the EPA/DHA groups experienced a greater reduction in TGs and a greater increase in HDL cholesterol, but also of total cholesterol and LDL cholesterol than ALA (Table 2). These findings agree with a meta-analysis of 6 RCTs lasting 1 y or longer, which reported that ALA supplementation had no significant beneficial (or negative) effect on blood lipid profile (Table 2) (60). Study duration has been shown to play an important role in lipid outcomes. A recent meta-analysis of 47 RCTs examined the effect of ALA intake on the lipid profile (66). Compared with control, dietary ALA significantly reduced total cholesterol, LDL cholesterol, and TGs (Table 2); however, the effect was attenuated in longer-term trials (65). This is consistent with the meta-analysis by Abdelhamid et al. (60), in which the greatest effect of ALA was in interventions lasting for 1–2 y compared with those longer than 2 y, which the authors attributed to a decrease in dietary compliance. Notably, in both Asian and European countries, ALA intake was associated with reductions in total cholesterol and LDL cholesterol, although the association was more pronounced in Asian countries (66). In an RCT with 100 patients diagnosed with nonalcoholic fatty liver disease, 30 g flaxseed daily plus positive lifestyle interventions for 12 wk significantly decreased total cholesterol, TGs, and LDL cholesterol, results that were not achieved in the positive lifestyle interventions–only group (67).

**TABLE 2:** Meta-analyses of randomized controlled trials of ALA for outcomes of cardiovascular disease risk markers^1^

Authors (ref.)	Outcomes assessed	Numbers in meta-analysis	Duration, wk	Source of ALA in treatment groups	ALA dose, g/d	Weighted mean differences (95% CIs) between intervention and control groups
Abdelhamid et al. (60)	Lipid profile	7 trials with 2201 participants	52–162	Variable (enriched margarine, flaxseed, walnuts)	1.9–4.5	Total cholesterol: −0.09 mmol/L (−0.23, 0.05 mmol/L)LDL-C: −0.05 mmol/L (−0.15, 0.04 mmol/L)HDL-C: −0.02 mmol/L (−0.08, 0.03 mmol/L)TGs: −0.03 mmol/L (−0.11, 0.05 mmol/L)
	BP	4 trials with 1671 participants				Systolic BP: −0.87 mm Hg (−4.48, 2.75 mm Hg)Diastolic BP: −1.42 mm Hg (−4.40, 1.57 mm Hg)
Pan et al. (64)	Lipid profile	28 trials with 1539 participants	2–52	Flaxseed (whole, ground), flaxseed oil, lignans	1.0–38	Total cholesterol: −0.10 mmol/L (−0.20, 0.00 mmol/L)LDL-C: −0.08 mmol/L (−0.16, 0.00 mmol/L)
Chen et al. (65)	Lipid profileGlucose	14 trials (with 3 arms: LCn–3FA, ALA, and placebo) with 1137 participants	2–24	Flaxseed oil (*n* = 6), linseed oil (*n* = 2), rapeseed oil (*n* = 1), mixed oils (*n* = 1), *Camelina sativa* oil (*n* = 1), botanical oil (*n* = 1), enriched margarine (*n* = 1)	1.9–10	ALA vs. placeboTotal cholesterol: −0.023 mmol/L (−0.164, 0.117 mmol/L)LDL-C: −0.098 mmol/L (−0.180, −0.016 mmol/L)HDL-C: 0.004 mmol/L (−0.023, 0.031 mmol/L)TGs: 0.051 mmol/L (−0.051, 0.154 mmol/L)Glucose: −0.101 mmol/L (−0.277, 0.076 mmol/L)ALA vs. LCn–3PUFAsTotal cholesterol: −0.179 mmol/L (−0.352, −0.006 mmol/L)LDL-C: −0.130 mmol/L (−0.253, −0.006 mmol/L)HDL-C: −0.033 mmol/L (−0.062, −0.004 mmol/L)TGs: 0.191 mmol/L (0.133, 0.249 mmol/L)Glucose: −0.072 mmol/L (−0.206, 0.061 mmol/L)
Yue et al. (66)	Lipid profile	47 trials with 2630 participants	3–104	Mainly flaxseed, walnuts, rapeseed, and derived oils	0.4–16	Total cholesterol: −0.140 mmol/L (−0.224, −0.056 mmol/L)LDL-C: −0.131 mmol/L (−0.191, −0.071 mmol/L)HDL-C: 0.008 mmol/L (−0.018, 0.034 mmol/L)TGs: −0.101 mmol/L (−0.158, −0.044 mmol/L)
Guasch-Ferré et al. (68)	Lipid profile	26 trials with 1059 participants	4–52	Walnuts	1.4–9.8	Total cholesterol: −0.181 mmol/L (−0.243, −0.119 mmol/L)LDL-C: −0.142 mmol/L (−0.199, −0.085 mmol/L)HDL-C: 0.003 mmol/L (−0.020, 0.025 mmol/L)TGs: −0.053 mmol/L (−0.101, −0.005 mmol/L)
	BP	8 trials with 363 participants				Systolic BP: −0.72 mm Hg (−2.75, 1.30 mm Hg)Diastolic BP: −0.10 mm Hg (−1.49, 1.30 mm Hg)
Sahebkar et al. (69)	Lipoprotein(a)	6 trials	6–52	Flaxseed products	1.4–9.3	Standardized mean difference: −0.22 (−0.41, −0.04)
Ursoniu et al. (71)	BP	15 trials with 1302 participants	4–52	Flaxseed products	1.2–15	Systolic BP: −2.85 mm Hg (−5.37, −0.33 mm Hg)Diastolic BP: −2.39 mm Hg (−3.78, −0.99 mm Hg)
Su et al. (81)	Inflammatory markers	25 trials with 2579 participants	4–104	Flaxseed oil (*n* = 15), rapeseed oil (*n* = 4), perilla oil (*n* = 2), enriched margarine (*n* = 2), mixed oils (*n* = 2)	1.0–14	Standardized mean differences:CRP: −0.06 (−0.24, 0.12)TNF-α: −0.03 (−0.36, 0.29)IL-6: −0.17 (−0.46, 0.12)sICAM-1: −0.06 (−0.26, 0.13)sVCAM-1: −0.24 (−0.56, 0.09)
Rahimlou et al. (85)	Inflammatory markers	32 trials with 1502 participants	2–12	Flaxseed products (whole or ground seed, oil, lignans)	Not reported	CRP: −0.34 mg/L (−0.89, 0.20 mg/L)hs-CRP: −0.75 mg/L (−1.19, −0.30 mg/L)TNF-α: −0.38 pg/mL (−0.75, −0.01 pg/mL)IL-6: −0.25 pg/mL (−0.70, 0.21 pg/mL)
Askarpour et al. (86)	Inflammatory markers	40 trials with 2520 participants	2–54	Flaxseed products (whole or ground seed, oil, lignans)	1–14	CRP: −0.387 mg/L (−0.653, −0.121 mg/L)TNF-α: −0.077 pg/mL (−0.317, 0.163 pg/mL)IL-6: −0.154 pg/mL (−0.299, −0.010 pg/mL)sICAM-1: −8.61 ng/mL (−21.94, 4.72 ng/mL)sVCAM-1: −22.81 ng/mL (−41.50, −4.12 ng/mL)E-selectin: −1.43 ng/mL (−4.07, 1.22 ng/mL)
Brown et al. (97)	Glycemic control	10 trials with 648 participants	24–52	Walnuts, flaxseed products, canola oil, rapeseed, and ALA-enriched margarines	2.2–9.5	Fasting glucose: −0.07 mmol/L (−0.16, 0.02 mmol/L)HbA1c: 0.01% (−0.43%, 0.45%)Fasting insulin: 5.3 pmol/L (−4.68, 15.27 pmol/L)HOMA-IR: 0.10 (−0.50, 0.70)
Neale et al. (98)	Glycemic control	16 trials	4 d–52 wk	Walnuts	1.4–5.9	Fasting glucose: 0.018 mg/dL (−0.046, 0.082 mg/dL)HbA1c: 0.03% (−0.00%, 0.06%)Fasting insulin: 0.21 pmol/L (−12.71, 13.13 pmol/L)HOMA-IR: −0.01 (−0.32, 0.30)
Jovanovski et al. (99)	Glycemic control	8 trials with 212 participants (type 2 diabetes)	8–52	Flaxseed oil (*n* = 4), walnuts (*n* = 3), chia seeds (*n* = 1)	1.5–7.4	Fasting glucose: 0.07 mmol/L (−0.61, 0.76 mmol/L)HbA1c: −0.01% (−0.32%, 0.31%)Fasting insulin: 7.03 pmol/L (−5.84, 19.89 pmol/L)
Mohammadi-Sartang et al. (122)	Adiposity measures	45 trials with 2561 participants	3–48	Flaxseed products (whole or ground seed, oil, lignans)	1–15.4	Body weight: −0.99 kg (−1.67, −0.31 kg)BMI: −0.30 kg/m^2^ (−0.53, −0.08 kg/m^2^)Waist circumference: −0.80 cm (−1.40, −0.20 cm)
Fang et al. (123)	Adiposity measures	27 trials with 2035 participants	2–104	Walnuts	1.5–10.8	Body weight: 0.08 kg (−0.03, 0.20 kg)BMI: −0.040 kg/m^2^ (−0.24, 0.16 kg/m^2^)Waist circumference: −0.19 cm (−1.03, 0.64 cm)Fat mass: 0.28% (−0.49%, 1.06%)

1 ALA, α-linolenic acid; BMI, body mass index; BP, blood pressure; CRP, C-reactive protein; HbA1c, glycated hemoglobin; HDL-C, HDL cholesterol; HOMA-IR, homeostatic model assessment of insulin resistance; hs-CRP, high-sensitivity C-reactive protein; IL-6, interleukin-6; LCn–3FA, long-chain n–3 fatty acid; LDL-C, LDL cholesterol; sICAM-1, soluble intercellular adhesion molecule-1; sVCAM-1, soluble vascular cell adhesion molecule-1; TG, triglyceride; TNFα, tumor necrosis factor-α.

The effects on lipids of walnuts, a relevant source of ALA, were examined in a 2018 meta-analysis of 26 RCTs (68). The pooled results indicated significant reductions of total cholesterol, LDL cholesterol, and TGs by walnut compared with control diets (Table 2). The lipid/lipoprotein effects of walnuts have been assessed recently in the largest and longest nut RCT to date, the Walnuts And Healthy Aging (WAHA) study (31). The WAHA trial allocated 708 healthy, independent-living participants, aged 63–79 y (68% women), residing in Barcelona, Spain, and Loma Linda, California, to usual diet supplemented with walnuts (15% of energy—about a half cup of walnuts) or control diet (usual diet with abstention from walnuts) for 2 y. Participants in the walnut group had significant reductions in total cholesterol (mean: −0.22 mmol/L), LDL cholesterol (mean: −0.11 mmol/L), and in their numbers of total LDL particles and small LDL particles, both risk factors for CVD.

Finally, according to a recent meta-analysis of RCTs (69), flaxseed products as sources of ALA appear to reduce lipoprotein(a) (Table 2), an LDL variant that increases the risk of CVD, particularly IHD, independently of LDL cholesterol.

#### BP

Several RCTs have evaluated the effect of ALA given on its own or as a component of foods on BP. In the FLAX-PAD RCT, participants with peripheral artery disease (75% hypertensive) consumed food products containing either 30 g milled flaxseed (treatment) or a combination of mixed dietary oils, milled wheat, and bran (control) for 6 mo. Flaxseed significantly reduced systolic BP and diastolic BP, with changes in ALA correlating with both endpoints (70). A meta-analysis of 15 RCTs using flaxseed supplements for outcomes of BP demonstrated significant reductions in both systolic and diastolic BP (Table 2). BP effects were greater in studies of ≥12 wk duration than in those lasting <12 wk (71). However, in a study conducted in 59 overweight/obese adults with prehypertension, no significant differences in BP assessed via 24-h ambulatory monitoring were observed after 12 wk of supplementation with 10 g refined cold-pressed flaxseed oil (providing 4.7 g ALA) or 10 g high-oleic sunflower oil (used as control) (72). Likewise, compared with the high-oleic oil control, flaxseed oil did not change brachial artery flow-mediated vasodilation, carotid-to-femoral pulse wave velocity, retinal microvascular calibers, and plasma markers of microvascular endothelial function during the fasting and postprandial phases (73).

The 2018 Guasch-Ferré et al. (68) meta-analysis of walnut RCTs reported null effects on BP (Table 2). However, most studies were small and of short duration. In a much larger and longer RCT, in a subsample of 236 participants in the WAHA trial (60% with mild hypertension) assessed with 24-h ambulatory BP monitoring, Domènech et al. (74) reported that mean office systolic BP decreased by 4.6 mm Hg (95% CI: −7.4, −1.8 mm Hg) in the walnut group, whereas participants in the upper tertile of baseline 24-h ambulatory systolic BP (>125 mm Hg) experienced a mean reduction of 8.5 mm Hg (95% CI: −12, −5.0 mm Hg) in systolic BP; no changes were observed in diastolic BP. In addition, participants in the walnut group required lower doses of antihypertensive drugs than control participants. The statistical power and long study duration with mostly hypertensive participants likely accounted for the BP-lowering effect of walnuts in the WAHA trial. Finally, in a study conducted in healthy Japanese participants, 6-mo supplementation with *Perilla frutescens* leaf powder (a source of ALA) significantly reduced systolic BP among subjects with baseline systolic BP ≥120 mm Hg (75).

#### Inflammation

Epidemiologic evidence has demonstrated inverse associations of dietary ALA and plasma inflammatory biomarkers. In a cross-sectional study within the Nurses’ Health Study I cohort (*n* = 727), ALA intake as assessed by FFQ was inversely related to plasma concentrations of C-reactive protein (CRP), IL-6, and E-selectin after controlling for multiple factors (76). In a cross-sectional study in Japan (*n* = 14,191) there was an inverse linear trend for CRP across quartile groups of ALA intake in men. In women, CRP concentrations in the lowest quartile of ALA intake were slightly elevated compared with the equivalent concentrations in the quartile 2 to quartile 4 intake categories (77). However, in another Japanese cross-sectional study with 1556 men and 1461 women aged 35–60 y, CRP was inversely related to ALA (*P* = 0.026) in women only (78).

In a cross-sectional substudy of 364 patients with CVD aged >45 y from a Brazilian cohort, dietary n–3 PUFA was inversely associated with markers of inflammation. Specifically, an increase in n–3 PUFA of 1 g/1000 kcal was associated with a 33% reduction in CRP and a 48% reduction in IL-1β (79). The study population consumed a typical Brazilian diet, low in marine food and high in vegetable oils, thus the total n–3 fraction evaluated was predominantly ALA; however, the precise amounts of ALA and LCn–3PUFAs are not known. In another cross-sectional analysis of the same cohort, a negative association between ALA and proinflammatory biomarkers was observed when comparing insulin-resistant and non–insulin resistant patients in secondary prevention of CVD (80).

RCTs have not consistently shown benefits of ALA on markers of inflammation. A systematic review and meta-analysis of 25 RCTs reported no significant effect of dietary ALA supplementation on circulating inflammatory markers (Table 2) (81). However, the authors noted that ALA supplementation decreased CRP in 2 studies of participants with high baseline CRP concentrations (>6 mg/L) (82, 83). Moreover, a negative linear relation was observed for CRP between the effect size and baseline CRP. These results suggest that ALA might decrease CRP in individuals with higher baseline CRP. A recent report from the sizable, 2-y WAHA trial reported benefits of the walnut diet at 15% of energy on markers of inflammation, with significant mean reductions ranging from 3.5% to 11.5% in granulocyte-monocyte colony stimulating factor, IFN-γ, IL-1β, IL-6, TNF-α, and E-selectin, although there was no effect on CRP (84).

A systematic review of 32 RCTs reported that consumption of flaxseed products led to a significant reduction of high-sensitivity CRP and TNF-α, but had no effect on standard CRP or IL-6 (Table 2) (85). A subgroup analysis revealed that changes were dependent on supplement type, BMI, and age, the latter being associated with a higher baseline inflammatory status. Thus, as would be expected, subjects with a higher BMI had significantly lower TNF-α and CRP with flaxseed supplementation. A subsequent systematic review and meta-analysis of 40 RCTs by Askarpour et al. (86) demonstrated improvements in some inflammatory biomarkers (Table 2). Reduced CRP concentrations were only observed for unhealthy or overweight participants and in trials that administered whole flaxseed and lignan supplement for ≥12 wk. Collectively, flaxseed supplementation in overweight/obese or high-risk participants with elevated inflammatory status has anti-inflammatory effects; however, confirmatory evidence is needed.

#### Markers of atherosclerosis

Several cross-sectional studies have evaluated the association between dietary or biomarker ALA and markers of atherosclerosis, such as intima–media thickness (IMT) and carotid and femoral artery plaques determined by ultrasonography, or calcified coronary plaques assessed by computed tomography. In US populations, ALA intake related inversely to carotid IMT and plaque (87) and to coronary artery plaques (88). ALA in serum phospholipids also related inversely to carotid IMT (89) and carotid and femoral plaque burden (90) in a cohort of Spanish dyslipidemic individuals. Another study from a Chinese cohort reported less thickening of carotid IMT and lower carotid plaque prevalence was associated with increasing ALA and DHA, but not EPA, in RBC membranes (91). Not all evidence, however, favors ALA for atherosclerosis outcomes. In 3314 participants in the Multi-Ethnic Study of Atherosclerosis, carotid plaque was assessed at baseline and after 9.5 y and related to plasma phospholipid fatty acids. ALA was unrelated to plaque prevalence or progression, whereas modest beneficial associations were observed for DHA (92). Similar results were reported from a large, prospective Chinese study (*n* = 4040) wherein carotid atherosclerosis was determined at baseline and after 8.8 y. Once again, no associations of ALA status in RBC membranes with IMT thickening or plaque prevalence/incidence were reported, whereas modest beneficial associations were observed for DHA (93). Finally, in a cross-sectional study conducted in patients undergoing carotid endarterectomy, RBC n–3 PUFAs were unrelated to the presence of symptomatic carotid disease (94).

The only RCT of ALA to examine changes in atherosclerosis markers was the MARGARIN study, conducted in 110 patients at high risk of IHD who were assigned a diet supplemented with margarine (80% fat, 60% of which as PUFAs) containing either 15% or 0.3% of total fat as ALA for 2 y. Results showed a similar progression rate of mean carotid and femoral IMT with the 2 interventions (95). However, small study size, no placebo control, and large between-individual variation in IMT changes limit the conclusion of this study. In a subcohort of the PREDIMED trial (*n* = 175), carotid plaque height was sonographically assessed at baseline and after intervention for a mean of 2.4 y. Compared with the control group (advice to follow a low-fat diet), participants consuming 30 g/d of mixed nuts (with 15 g walnuts, which provided ∼1.3 g ALA) showed regression of maximum plaque height (96).

### ALA and cardiometabolic risk markers

#### Glycemic control

A meta-analysis of 83 RCTs of ≥24 wk duration assessed effects of increasing PUFA intake on diabetes diagnoses, fasting glucose, fasting insulin, glycated hemoglobin (HbA1c), and/or HOMA-IR (97). The pooled results indicated that intake of ALA, assessed in 12 trials, had little or no effect on T2D risk or glycemic control (Table 2). Null results on risk of incident T2D or glycemic control were reported in RCTs of LCn–3PUFAs. A similar lack of effect of both ALA and LCn–3PUFAs on fasting glucose was described in the recent meta-analysis of Chen et al. (65) (Table 2).

Likewise, the 2018 meta-analysis by Guasch-Ferré et al. (68) reported little effect of walnut diets on markers of glycemia and insulinemia in 6 RCTs. A more recent meta-analysis of 16 RCTs that assessed effects of walnut diets on biomarkers of glycemic control did not find any benefit (Table 2) (98).

More relevant than effects of n–3 PUFAs on glycemic markers of nondiabetic individuals are effects in patients with T2D. A 2017 meta-analysis of 8 RCTs also found a neutral effect of ALA intake on biomarkers of glycemic control in this population, such as fasting blood glucose, HbA1c, or fasting blood insulin concentrations (Table 2) (99). It must be emphasized that participants in these RCTs received optimal antidiabetic drug treatment and had well-controlled T2D (mean HbA1c = 6.8%), which limits the possibility of further improving glycemic control by diet. The authors noted, however, that ALA dose was correlated with reductions in fasting blood glucose and HbA1c. The lack of effect of LCn–3PUFAs on glycemic control among patients with T2D was confirmed in a 2020 meta-analysis of 12 RCTs using various doses of fish oil (100).

#### T2D

The association between intake of n–3 PUFAs and the risk of T2D has been assessed in several epidemiologic studies. A 2012 meta-analysis of prospective studies examined the risk associated with dietary ALA in 7 cohorts and ALA as a biomarker in 6 cohorts (101). Although nonsignificant, there was a trend toward T2D risk reduction with both dietary and biomarker ALA (Table 1). Neither consumption of fish/seafood or EPA + DHA nor EPA + DHA biomarkers were significantly associated with T2D risk. These findings are supported by a recent dose-response meta-analysis of 23 observational studies with fatty acid intake data in relation to incident T2D (102). In linear dose-response analyses, ALA intake was unrelated to T2D risk (Table 1), but a nearly significant nonlinear dose–response association with decreased T2D risk was found for ALA intakes ≤560 mg/d, with an RR of 0.95 (95% CI: 0.90, 1.00). Concerning LCn–3PUFAs, there was a direct association with T2D risk that was approximately linear for intakes ≤270 mg/d for the overall studies, but risk decreased with increasing intake in Asian populations whereas it increased in US cohorts. A recent analysis of 20 prospective studies from 14 countries participating in the Fatty Acids and Outcomes Research Consortium (FORCE) examined marine- or plant-derived biomarkers of n–3 PUFAs in relation to incident T2D (103). ALA was not significantly associated with T2D (Table 1). However, when considering the blood compartment where fatty acids were analyzed, there was a significant inverse association between plasma phospholipid ALA and T2D, based on 9 cohorts. In the overall analyses, higher biomarker EPA, DPA, DHA, and their sum were associated with lower T2D incidence, with decreases ranging from 8% to 21%.

The Nurses’ Health Study and Health Professionals Follow-Up Study cohorts found that consumption of walnuts as a source of ALA was inversely related to T2D risk (RR: 0.76; 95% CI: 0.62, 0.94) (104), whereas another large cross-sectional study in the United States reported an even greater benefit of walnuts on T2D (RR: 0.47; 95% CI: 0.31, 0.71) (105).

#### MetS

Numerous epidemiologic studies have examined the association of n–3 PUFAs, including ALA, with MetS. A meta-analysis of 27 cross-sectional and case-control studies conducted between 2005 and 2016 investigated biomarker n–3 PUFAs in relation to MetS prevalence (106); 15 studies considering ALA independently of other n–3 PUFAs were included. Whereas higher total circulating n–3 PUFAs were associated with lower MetS risk (pooled OR: 0.63; 95% CI: 0.49, 0.81), no association was found for ALA (Table 1). Likewise, there were no differences in circulating ALA between cases and controls. Similar conclusions were reached in a recent meta-analysis of epidemiologic studies conducted between 2010 and 2016 (107), mostly using biomarker ALA (Table 1). Other studies not included in these meta-analyses have evaluated the association between ALA and MetS. A cross-sectional study using adipose tissue fatty acids found low adipose ALA and high adipose EPA were related to a higher frequency of MetS in Mesoamerican adults, but not in their children (108). Divergent results were reported by a prospective analysis (*n* = 2754) in the Chinese Nutrition and Health Study in which RBC PUFAs at baseline were related to incidence of MetS and its components after 8.8 y of follow-up. Increasing ALA was associated with higher MetS rates and hypertriglyceridemia, whereas increasing EPA and DHA were related to lower incidence of MetS and its components high TG, low HDL cholesterol, and high BP (109). These results are consistent with a recently completed prospective examination of 1245 Chinese adults with baseline measurements of RBC PUFAs that assessed MetS incidence after a 6-y follow-up (110). Although this study focused on n–6 PUFAs and acylcarnitines, a slightly increased risk of incident MetS for ALA (RR for 1-SD increment: 1.09; 95% CI: 1.03, 1.15) and no significant associations for total n–3 PUFAs or other n–3 PUFAs were reported; ALA was also positively associated with increased TGs and inversely associated with HDL cholesterol. In contrast, a recent cross-sectional study in a large Spanish cohort (*n* = 6560) of older MetS patients found no association of dietary ALA with MetS criteria (111).

No RCTs have tested intake of isolated ALA for outcomes of MetS components and few have used ALA-enriched diets. In a 3-arm, 12-wk RCT conducted among 283 Chinese participants with MetS, lifestyle counseling alone or associated with flaxseed (30 g/d) or walnuts (30 g/d) led to similar MetS reversion rates of 21%–27%; however, the reversion rate of central obesity was higher in the flaxseed (19%) and walnut (16%) groups than in the lifestyle-alone group (6%) (112). A low-calorie rapeseed oil diet enriched with ALA (providing 3.5 g/d) was compared with an ALA-poor olive oil diet over 6 mo to assess effects on cardiometabolic risk factors in MetS patients; cardiometabolic factors decreased with both diets, but the ALA diet further reduced TGs and diastolic BP (113). In the WAHA trial, walnut supplementation at 15% of energy for 2 y in a healthy elderly population had no effect on MetS risk (114).

#### Obesity

Evidence suggests that the type of fat consumed influences how it is partitioned, i.e., for either energy or storage. In particular, the degree of long-chain fatty acid unsaturation is proposed to influence the direction of dietary fat toward immediate oxidation or storage. Thus, the quality of fat consumed may become important for long-term weight management. Human studies have demonstrated a pattern of selective oxidation of long-chain unsaturated fatty acids compared with long-chain SFAs (115, 116). However, differences among the various unsaturated fatty acids are less clear. In a study in which human subjects were given a labeled fatty acid in a blended meal, DeLany et al. (117) found that the oxidation of linolenate was higher than that of linoleate and oleate, whereas the latter 2 had similar rates of oxidation. In contrast, McCloy et al. (118) showed that the oxidation of ^13^C-linoleate was lower than that of oleate and linolenate. Thus, because of these inconsistencies it is not clear what the effects of ALA are on energy metabolism in humans.

Few epidemiologic studies have examined adiposity measures in relation to ALA intake. In a cross-sectional study of baseline data of 6560 participants in the PREDIMED-Plus trial, BMI (in kg/m^2^) was significantly lower in the highest than in the lowest quintile of total PUFAs (*P* < 0.001), linoleic acid (LA) (*P* = 0.020), and ALA (*P* < 0.001) (107). In a study by Liu et al. (119), the impact of changes in intakes of varying types of dietary fat on long-term weight gain was investigated every 4 y over a 20- to 24-y follow-up period in 3 independent prospective cohorts. The results demonstrated an inverse association between n–6 PUFA intake and weight gain that was mainly driven by LA, as well as an inverse association between n–3 PUFAs and weight gain that was driven by n–3 PUFAs of both marine (EPA and DHA) and plant origin (ALA). In evaluating these studies, it is possible that plant-based PUFAs may modulate human obesity more than marine-derived PUFAs do. In a Danish study among 1212 adults, intake of total n–3 PUFAs showed an inverse association with adiposity measures (BMI, waist circumference, and fat mass), which persisted only for ALA (not EPA or DHA) after adjusting for potential confounders (120). Similarly, analysis of data from the sizable Heidelberg cohort of the European Prospective Investigation into Cancer and Nutrition study, with a mean follow-up time of 6.5 y, also reported an inverse association between dietary ALA intake (but not EPA and DHA) and weight gain in women (121).

Many RCTs have assessed changes of body weight and other adiposity measures after intake of ALA-rich foods, usually as secondary outcomes of studies focusing on changes in lipids and other CVD risk factors. The effects of RCTs using either flaxseed products or walnuts on adiposity markers have been summarized in 2 meta-analyses (122, 123) (Table 2). Of note, flaxseed interventions were associated with small but favorable adiposity outcomes, whereas walnuts had neutral effects.

### ALA and cognition

CVD and cognitive decline (in particular Alzheimer disease) share similarities in terms of their risk factors and underlying pathology. Both their epidemiologic and genetic risk factors overlap significantly. In addition, both present with a protracted asymptomatic stage, often with presence of specific pathological changes in otherwise asymptomatic individuals. This is the basis for the conjecture that prevention of cognitive decline can benefit from the knowledge and existing lifestyle interventions proven to be efficacious in the cardiovascular field. n–3 PUFAs supplied by fatty fish (in particular DHA) are a paradigmatic case of “good for the heart, good for the brain” because, in addition to the long-known cardiovascular benefits of regular DHA intake, there is increasing observational evidence for salutary effects of DHA in Alzheimer disease (124). For a long time, brain benefits of dietary ALA were mostly ascribed to the marginal conversion of ALA to DHA (125). However, experimental evidence on brain effects of ALA [reviewed in Devassy et al. (126)] fostered interest in whether sustained ALA intake might promote resilience against cognitive decline and dementia independently, irrespective of whether the diet provides sufficient amounts of DHA.

Observational evidence for ALA is scarce compared with DHA. In a longitudinal study the association between n–3 PUFA intake and 5-y cognitive change was assessed in 915 older individuals. The authors reported that higher ALA intake was associated with slower global cognitive decline, but only in participants carrying an apoE*-ε4* allele (127). This methodological approach was used in the prospective Doetinchem Cohort Study (*n* = 2612 participants aged 43–70 y at baseline), which reported that higher n–3 PUFA (especially ALA) intake, but not EPA and DHA, was associated with slower decline in global cognitive function and memory (128). Regarding MRI-assessed brain outcomes, in a cross-sectional study of 672 cognitively unimpaired participants (aged 80 y), higher ALA intake was associated with larger cortical thickness (129). In relation to incident dementia, results are inconclusive. Two longitudinal studies reported that ALA was associated with a decreased risk of dementia (130, 131). In contrast, another 2 studies reported a significantly lower risk of dementia was associated with circulating EPA, but not ALA (132, 133).

The AlphaOmega trial, designed primarily as a cardiovascular prevention study, tested the effects of ALA on cognitive impairment as a secondary endpoint in 2911 stable coronary patients (aged 60–80 y). After a median of 3.3 y, no significant differences in cognitive decline were observed for the active treatment groups (1.9 g/d of ALA, 400 mg/d of EPA + DHA, or both) compared with placebo (134). However, ALA supplementation was associated with somewhat better results than EPA + DHA, because it significantly reduced cognitive decline in patients aged <70 y and in those with fish intake >20 g/d and reduced the risk of severe cognitive decline by ∼10%, although without reaching statistical significance. However, the Mini-Mental State Examination, an approximate global measure of cognitive function, was the only neuropsychological test administered, which dictates caution in the interpretation of these findings. Finally, the WAHA trial examined 2-y changes of cognitive function, assessed with a battery of neuropsychological tests, after a diet enriched with walnuts at ∼15% energy (30–60 g/d) compared with a control diet (abstention from walnuts) in 708 free-living cognitively unimpaired elders from 2 different sites (Barcelona, Spain and Loma Linda, California). Although walnut supplementation did not delay cognitive decline, post hoc analyses showed that participants from the Barcelona site in the walnut diet arm, but not those from Loma Linda, improved global cognition and perception scores compared with controls. It must be noted that Barcelona participants were more at risk of cognitive decline than their Loma Linda counterparts owing to less education and more smoking. A benefit from the intervention in the Barcelona cohort was also suggested by findings from brain fMRI performed in a randomly selected subset of participants (135).

## Conclusions and Recommendations on ALA Intake

Although the evidence for the benefits of ALA on CVD, the lipid/lipoprotein profile, BP, systemic inflammation, and cognition is increasing, it is also apparent that the variable results reported reflect different study designs, endpoints, how ALA was consumed and the amount, as well as for how long, the background diet, and the study participants. For CVD, there is some evidence from both epidemiologic studies and RCTs of benefits of ALA at higher intakes (>2 g/d; 0.6–1% of energy), yet the evidence is disparate, and the positive evidence is somewhat confusing, making it daunting to understand the effects of ALA. For example, there are benefits of ALA when LCn–3PUFAs are low and also benefits when intakes of both ALA and LCn–3PUFAs are high. These positive results warrant further study to clarify our understanding of the role that ALA plays in CVD prevention and treatment. Nonetheless, the research does show beneficial effects of ALA on reducing atherogenic lipids and lipoproteins, BP, and markers of inflammation, which provides the biological basis to explain the CVD benefits and pursue further research. With respect to T2D, MetS, and obesity, the evidence is inconclusive for benefits of ALA. There are some exciting new findings suggesting benefits of ALA on cognition but much more research is needed before meaningful conclusions can be reached.

**FIGURE 1:**
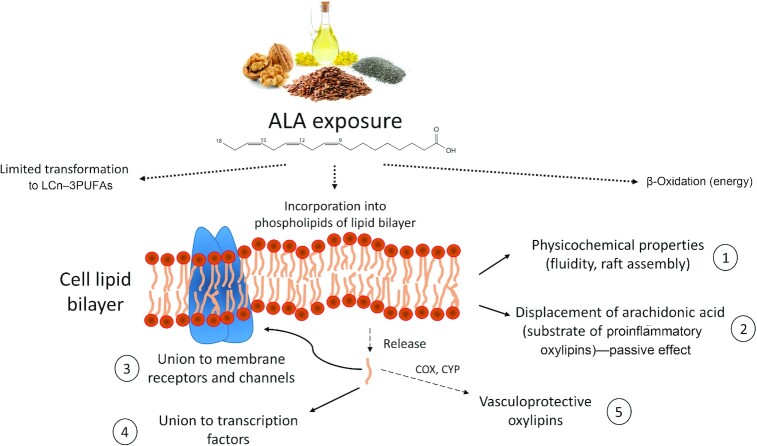
Proposed primary mechanisms underlying the benefits of dietary ALA. Dietary ALA (obtained from flaxseed, walnuts, chia seeds, canola oil—see Table 3 for detailed information on dietary sources) undergoes fatty acid β-oxidation, conversion to LCn–3PUFAs, and incorporation into cell membrane phospholipids. As occurs with other dietary PUFAs, ALA incorporation into a membrane alters the biophysical membrane properties (1), and partially displaces arachidonic acid, a substrate of proinflammatory lipid mediators (2). Once cleaved from membranes by the action of phospholipases, ALA binds to specific transmembrane proteins or voltage-gated channels (3), promotes/inhibits gene expression after binding transcription factors (4), and might be converted to anti-inflammatory and antihypertensive lipid mediators by the action of COX and CYP (5). ALA, α-linolenic acid; COX, cyclooxygenase; CYP, cytochrome P450; LCn–3PUFA, long-chain n–3 PUFA.

Experimental research has provided insight into mechanisms underlying ALA effects (**Figure 1**), some of which are related to conversion of ALA to its longer-chain counterparts. However, ALA also displays intrinsic effects. Besides modulating cardiac ion channels (136), ALA is converted to oxylipins that contribute to vascular health by reducing inflammation and improving blood pressure (137). Although there remain questions about the benefits of ALA on health, the evidence at this juncture supports current dietary guidance, which states that to achieve nutritional adequacy, ALA should provide 0.6%–1.0% of total energy or 1.1 g/d for women and 1.6 g/d for men (6). ALA is abundant in various plant foods, including walnuts, flaxseeds, and cooking oils, such as canola (rapeseed) oil, soybean oil, and flaxseed oil (**Table 3**). However, most of these foods are rich in bioactive molecules other than ALA, which contribute to the overall effect. The paucity of RCTs testing ALA on its own (Table 2) makes it difficult to disentangle the exact contribution of ALA, particularly if the different food components interplay, as actually occurs. In any case, food sources high in ALA should be included as part of a heart-healthy dietary pattern. As the results of many of the studies described here indicate, ALA is essential with many potential therapeutic properties. Nevertheless, additional well-designed studies are needed in order to establish unequivocal support to evolve dietary ALA recommendations beyond the current Adequate Intake (for achieving nutrient adequacy) to a DRI recommendation for chronic disease risk reduction.

**TABLE 3:** ALA content of selected foods, and daily servings needed to meet adequate intakes^1^

Food source of ALA^2^	Amount of ALA per serving,^3^ g	Servings per day needed by men to meet recommendation (1.6 g ALA/d)	Servings per day needed by women to meet recommendation (1.1 g ALA/d)
Pumpkin seeds	0.03	53.33	36.67
Olive oil	0.10	16.00	11.00
Edamame beans	0.28	5.71	3.93
Soybean oil	0.95	1.68	1.16
Canola oil	1.28	1.25	0.86
Walnut oil	1.41	1.13	0.78
Walnuts, English	2.57	0.62	0.43
Camelina seed oil^4^	4.49	0.35	0.24
Chia seeds	5.05	0.32	0.22
Flaxseeds, whole	6.46	0.25	0.17
Flaxseeds, ground	6.55	0.24	0.17
Flaxseed oil	7.26	0.22	0.15
Perilla seed oil^5^	8.16	0.20	0.13

1 ALA, α-linolenic acid.

2 Unless otherwise stated, source is USDA ARS (138).

3 Serving: oil = 1 tbsp (13.6 g); seeds/nuts = 1 oz (28.35 g); edamame = ½ cup (77.5 g).

4 Data from Ergönül and Özbek (139).

5 Data from Bondioli et al. (140).

## Notes

Supported by a California Walnut Commission grant. The funding agency had no involvement in the study design, data collection, analyses, interpretation of the data, or writing of the manuscript.

Author disclosures: AS-V, PK-E, and ER have received research funding through their institutions from the California Walnut Commission (CWC), Folsom, CA. AS-V has received support from the CWC to attend professional meetings. PK-E and ER were nonpaid members of the Scientific Advisory Council of the CWC. ER was a paid member of the CWC Health Research Advisory Group and has received personal money from the CWC for presentations. The other author reports no conflicts of interest.

Abbreviations used: AF, atrial fibrillation; ALA, α-linolenic acid; BP, blood pressure; CRP, C-reactive protein; CVD, cardiovascular disease; DPA, docosapentaenoic acid; HbA1c, glycated hemoglobin; HF, heart failure; IHD, ischemic heart disease; IMT, intima–media thickness; LCn–3PUFA, long-chain n–3 PUFA; MetS, metabolic syndrome; MI, myocardial infarction; PREDIMED, Prevención con Dieta Mediterránea; RCT, randomized controlled trial; TG, triglyceride; T2D, type 2 diabetes; WAHA, Walnuts And Healthy Aging.
